# Early characterization of an adult population at an insurer’s point of entry as an opportunity to identify hospitalization risk

**DOI:** 10.15649/cuidarte.3290

**Published:** 2024-05-28

**Authors:** Lorena María Vargas-Díaz, Olga Patricia Pachón Arciniegas, Santiago Osorio Rojas, Edgar Fabián Manrique-Hernández, Anderson Bermon Angarita

**Affiliations:** 1 Fundación Cardiovascular de Colombia, Bucaramanga, Colombia. lorenavar@gmail.com Fundación Cardiovascular de Colombia Bucaramanga Colombia lorenavar@gmail.com; 2 Fundación Cardiovascular de Colombia, Bucaramanga, Colombia. patriciapachon@fcv.org Fundación Cardiovascular de Colombia Bucaramanga Colombia patriciapachon@fcv.org; 3 Fundación Cardiovascular de Colombia, Bucaramanga, Colombia. rojasosorio43@gmail.com Fundación Cardiovascular de Colombia Bucaramanga Colombia rojasosorio43@gmail.com; 4 Fundación Cardiovascular de Colombia, Bucaramanga, Colombia. fabianmh1993@gmail.com Fundación Cardiovascular de Colombia Bucaramanga Colombia fabianmh1993@gmail.com; 5 Fundación Cardiovascular de Colombia, Bucaramanga, Colombia. andersonbermon@fcv.org Fundación Cardiovascular de Colombia Bucaramanga Colombia andersonbermon@fcv.org

**Keywords:** Health Profile, Insurance Health, Health Management, Perfil de Salud, Seguro de Salud, Gestión en Salud, Perfil de Saúde, Seguro Saúde, Gestão em Saúde

## Abstract

**Introduction:**

Health Benefit Plan Administrators must manage the health risk of their members. Therefore, health characterization is performed from enrollment to support decision-making and timely intervention.

**Objective:**

To analyze the historical results of characterizing the adult population on admission to the insurance company in relation to the demand for all-cause and psychiatric hospitalization services.

**Materials and Methods:**

An observational cross-sectional studywith members over 18 years of age, in which an analysis was made of the characterization of the adult population of the insurer and its association with the use of medicalconsultationservicesinprimarycareandall-causeandpsychiatric hospitalizations. Bivariate and multivariate analysis was made, and odds ratios (OR) were calculated in logistic regression.

**Results:**

Variables significantly associated with having an all-cause hospitalization were identified: having referred history of heart disease OR=1.71(95%CI: 1.33; 2.20), respiratory disease OR= 1. 30(95%CI: 1.04; 1.61), chronic kidney disease OR=1.66(95%CI: 1.13; 2.45), cancer OR=1.65(95%CI: 1.14; 2.40), taking any medication permanently OR=1.35(95%CI: 1.174; 1.56) and smoking OR=1.44(95%CI: 1.12; 1.85). For psychiatric hospitalizations, a history of discouragement, depression, or little hope was relevant with OR=5.12(95%CI: 1.89; 13.87).

**Discussion:**

The characterization of patients during enrolment allowed the identification of predictor variables of hospitalization, guiding management from the primary care level minimizing costs and catastrophic health events.

**Conclusion:**

The timely identification of specific patient profiles allows timely actions to minimize health costs and catastrophic health events.

## Resumen

**Introducción:**

Las Entidades Administradoras de Planes de Beneficios deben gestionar el riesgo en salud de sus afiliados. Por tanto, desde la afiliación se realiza la caracterización en salud para apoyar la toma de decisiones e intervención oportuna.

**Objetivo:**

Analizar los resultados históricos de la caracterización de población adulta al ingreso de la aseguradora en relación con la demanda de servicios de hospitalización por causa general y patología psiquiátrica.

**Materiales y Métodos:**

Estudio observacional de corte trasversal con los afiliados mayores de 18 años en donde se realizó análisis de la caracterización de la población adulta y su asociación con el uso de consultas médicas en Atención Primaria y hospitalizaciones por todas las causas y por patología psiquiátrica. Se realizaron análisis bivariado y multivariado y se calcularon OR (Odds Ratio) en regresión logística.

**Resultados:**

Se identificaron variables asociadas significativamente con hospitalización por cualquier causa: haber referido antecedente de enfermedad cardiaca OR=1,71(IC95%: 1,33; 2,20), enfermedad respiratoria OR= 1,30(IC95%: 1,04; 1,61), enfermedad renal crónica OR=1,66(IC95%: 1,13; 2,45), cáncer OR=1,65(IC95%: 1,14; 2,40), tomar algún medicamento de forma permanente OR=1,35(IC95%: 1,174; 1,56) y fumar OR=1,44(IC95%: 1,12; 1,85). Para hospitalización psiquiátrica fue relevante el antecedente de desánimo, depresión o poca esperanza OR=5,12(IC95%: 1,89; 13,87).

**Discusión:**

La caracterización realizada a los pacientes en la afiliación permitió identificar variables predictoras de hospitalización orientando la gestión desde el nivel primario, minimizando costos y eventos catastróficos en salud.

**Conclusión:**

La identificación oportuna de perfiles específicos de pacientes permite realizar acciones oportunas para minimizar costos sanitarios y eventos catastróficos en salud.

## Resumo

**Introdução:**

As Entidades Administrativas de Planos de Benefícios devem gerenciar o risco à saúde de seus associados. Portanto, a partir da filiação é realizada a caracterização da saúde para apoiar a tomada de decisão e a intervenção oportuna.

**Objetivo:**

Analisar os resultados históricos da caracterização da população adulta no momento da admissão na seguradora em relação à procura por serviços de internação por causas gerais e patologia psiquiátrica.

**Materiais e Métodos:**

Estudo observacional transversal com membros maiores de 18 anos onde foi realizada análise da caracterização da população adulta e sua associação com a utilização de consultas médicas na Atenção Básica e internações por todas as causas e por patologia psiquiátrica. Foram realizadas análises bivariadas e multivariadas e calculado OR (Odds Ratio) em regressão logística.

**Resultados:**

Foram identificadas variáveis significativamente associadas à internação por qualquer causa: ter relatado história de doença cardíaca OR=1,71(IC95%: 1,33; 2,20), doença respiratória OR= 1,30(IC95%: 1,04; 1,61), doença renal crônica doença OR= 1,66 (IC 95%: 1,13; 2,45), câncer OR=1,65(IC 95%: 1,14; 2,40), tomar algum medicamento permanentemente OR=1,35(IC 95%: 1,174; 1,56) e fumar OR=1,44 (IC 95%: 1,12; 1,85). Para internação psiquiátrica foi relevante história de desânimo, depressão ou baixa esperança, OR=5,12 (IC 95%: 1,89; 13,87).

**Discussão:**

A caracterização realizada aos pacientes na afiliação permitiu identificar variáveis preditoras de internação, orientando o manejo desde o nível primário, minimizando custos e eventos catastróficos de saúde.

**Conclusão:**

A identificação oportuna de perfis específicos de pacientes permite que ações oportunas sejam tomadas para minimizar custos de saúde e eventos catastróficos de saúde.

## Introduction

Within the Comprehensive Health Care Policy, the Health Benefit Plan Administrators (EAPB, for its Spanish acronym) have a fundamental role in managing the health risk of their members. They are responsible for identifying, assessing, measuring, treating, following up, and monitoring the risks of their insured population^1^. To manage risks in an appropriate and timely manner, the EAPB conducts a process of health characterization of its members to identify and implement early interventions for identified risks and to support the decision-making process in providing primary healthcare services.

Avoidable hospitalizations should be minimized to protect members’health and reduce healthcare costs. Precursors of a higher risk of hospitalization have been identified, such as advanced age, living alone, dependence in activities of daily living^2^^,^^3^, and cognitive, visual, communication, and gait problems that hinder patients’ autonomy and self-care and have been shown to increase the risk of hospitalization significantly^4^^,^^5^.

In addition, most avoidable hospitalizations occur in people over the age of 65 who have more comorbidities^6^, and it has been documented that the greater the number of diagnoses, the greater the probability of hospitalization^5^^,^^7^; therefore, primary care providers need to be aware of this evidence for risk management. Studies have been conducted to identify the population most at risk for hospitalization or emergency department visits^8^, and instruments have been designed to analyze the hospitalization risk in specific patient populations. These studies considered the number of diseases and their severity to classify the risk of hospitalization^9^ and considered the importance of investigating patients’ health self-assessment as an additional predictor^10^.

The objective of the present research was to analyze the historical results of the characterization of members who enrolled in EAPB in the metropolitan area of Bucaramanga and were over 18 years of age. The analysis was related to the demand for hospitalization services and primary healthcare from 2018 to 2021 to identify the members’ risk of all-cause and psychiatric hospitalizations.

## Materials and Methods

A cross-sectional observational study was conducted with members over 18 years of age enrolled from 2018 to 2021 with the EAPB, whose niche is the metropolitan area of Bucaramanga. The EAPB, established as a health insurance company, commenced operations on September 1, 2018^11^. As part of the enrollment process, the insurer conducts a health characterization or profiling of the applicants at the time of enrollment with the goal of early risk identification and intervention by the primary healthcare service provider. The data is collected at the moment of enrollment, or the new member is contacted later to obtain the data. However, it is not possible to characterize all members because not all enrollments are made through the insurer’s functionaries but through the Transactional Enrollment System or through mass enrollments, such as when patients are transferred from liquidated health insurance companies and directly assigned by health authorities to other companies, or because applicants do not provide the correct contact information at the time of enrollment. No sampling was performed for this analysis, as the data available from the characterizations made to the members at the time of application to the EAPB were used.

The characterization survey used is an instrument designed by the EABP. The survey asks about medical and family history, dietary habits, physical activity, substance use, and anthropometric measurements.

The aim is to identify at an early stage the risk factors or behaviors that could lead to health problems for the members and their families and, based on each response, to create a process of induced demand for the various programs offered by the EAPB. Algorithms are applied to the results of the characterization survey to identify the health promotion and maintenance programs for each profiled member and are sent to the primary care institution for management.

This study used data from medical consultations at primary care institutions and hospitals. In this analysis, we sought to identify variables from the characterization survey applied to members that could be related to all-cause and psychiatric hospitalizations; this segmentation is made considering that these events are attended in different institutions. The information from the sources was registered in Microsoft Excel and, after validation, exported to and analyzed in Stata/SE version 17. Mendeley Data was used to store the consolidated database^12^.

Categorical variables were described using absolute and relative frequencies, and continuous variables were described using central tendency and dispersion measures. Pearson’s chi-squared and Fisher’s exact tests were used to determine associations between qualitative variables. The distribution of continuous variables was determined using the Kolmogorov-Smirnov and Mann-Whitney U tests for differences between medians, as the normality assumption was not met.

For the multivariate analysis, the variables that in the bivariate analysis showed an association with having a hospital event for any cause or psychiatric reasons were included, and a logistic regression model was constructed with which the OR, 95% confidence intervals, and p-value were calculated. The cut-off point was set at p-value <0.05.

The Ethics Committee of the Fundación Cardiovascular de Colombia approved this research. According to Resolution 0084 of 1993, this study is classified as research without risk because the information was collected through anonymized secondary data, and no intervention was performed on individuals or populations that could pose a risk to their physical, mental, or social integrity.

## Results

**Figure 1:**
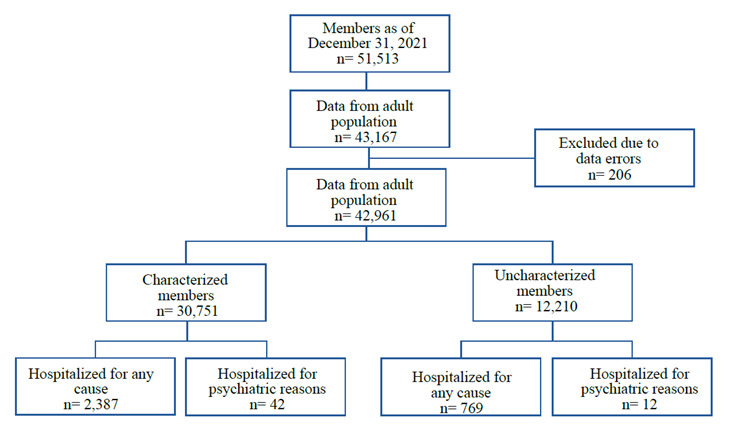
Data flow chart

Data were obtained from 43,162 members over 18 years of age enrolled in the insurance company as of December 31, 2021, and 206 records were discarded due to data entry errors. A total of 42,961 members were included in the analysis, of which 30,751 (71.58%) had results from the characterization survey (Figure 1).

Differences were found in age, use of first-level outpatient services, psychiatric care, and all-cause hospitalization among the population, with greater use of these services among characterized members than uncharacterized members. Similarly, the median age was lower among those who were characterized, and these differences were statistically significant (p<0.001). No differences were found between those with and without characterization regarding sex and hospitalization for psychiatric illness (Table 1).

**Table 1:** Age, sex, and service use characteristics of members according to the characterization

Variable	Characterized members n=30,751	Uncharacterized members n=12,210	^p^
Enrollment age (years)			<0.001
Minimum - Maximum	18 - 100	18 - 109	
Median (IQR)	37.52 (23-85)	42.34 (25-85)	
Sex			0.137
Female	16,247 (52.84)	6,549 (53.64)	
Male	14,499 (47.16)	5,661 (46.36)	
First-level outpatient consultation	20,366 (66.23)	6,257 (51.24)	<0.001
Outpatient psychiatry consultation	615 (2.00)	170 (1.39)	<0.001
All-cause hospitalization	2,387 (7.76)	769 (6.30)	<0.001
Hospitalized for psychiatric illness	42 (0.14)	12 (0.10)	0.312

IQR: Interquartile range. P-value: Qualitative variables with Pearson’s chi-squared test, numerical variables with Mann-Whitney U test.

In analyzing the questions in the characterization survey, an initial bivariate analysis was conducted to ascertain any relationship between the dependent variables (those found in the characterization survey) and hospital events’ outcomes. In this regard, an association was found between having an all cause hospitalization and self- reported conditions such as diabetes, hypertension, stroke, heart disease, dyslipidemia, respiratory disease, chronic kidney disease, cancer, history of occupational disease, regular intake of medication for high blood pressure or other condition, family history of diabetes, current smoking status, presence of any physical disability, or feelings of discouragement, depression, or little hope within the past 30 days, or diminished interest or pleasure in activities usually enjoyed and being overweight or obese. Similarly, doing any physical activity was associated with a lower risk of all cause hospitalization, as was having reported alcohol consumption. For hospitalization for psychiatric reasons, an association was found between reporting feelings of discouragement, depression, or little hope within the past 30 days, or having little interest or pleasure in activities usually enjoyed, and the use of psychoactive substances (Table 2).

**Table 2:** Self-reported characteristics associated with all-cause and psychiatric hospitalizations

Variable	All-cause hospitalization					Hospitalization for psychiatric reasons				
	No (28,364)	Yes (2,387)	OR	95% CI	^p^	No (30,709)	Yes (42)	OR	95% CI	^p^
Personal background n (%)									
Diabetes	1,030 (3.63)	133 (5.57)	1.57	1.30 - 1.89	<0.001	1,162 (3.78)	1 (2.38)	0.62	0.09 - 4.51	0.637
High blood pressure	3,028 (10.68)	360 (15.08)	1.49	1.32 - 1.67	<0.001	3,385 (11.02)	3 (7.14)	0.62	0.19 - 2.01	0.427
Stroke or thrombosis	144 (0.51)	31(1.30)	2.58	1.75 - 3.81	<0.001	175 (0.57)	0	-	-	-
Heart disease	630 (2.22)	126 (5.28)	2.45	2.02 - 2.98	<0.001	755 (2.46)	1 (2.38)	0.97	0.13 - 7.04	0.974
Dyslipidemia	2,958 (10.43)	293 (12.27)	1.20	1.06 - 1.37	0.005	3,248 (10.58)	3 (7.14)	0.65	0.20 - 2.10	0.473
HIV/AIDS	42 (0.15)	3 (0,13)	0.85	0.26 - 2.74	0.847	45 (0.15)	0	-	-	-
Respiratory disease	1,147 (4.04)	136 (5.70)	1.43	1.19 - 1.72	<0.001	1,282 (4.17)	1 (2.38)	0.56	0.77 - 4.07	0.567
Chronic kidney disease	261 (0.92)	52 (2.18)	2.38	1.76 - 3.24	<0.001	313 (1.02)	0	-	-	-
Cancer	258 (0.91)	42 (1.76)	1.95	1.40 - 2.71	<0.001	300 (0.98)	0	-	-	-
Occupational disease	300 (1.06)	37 (1.55%)	1.47	1.04 - 2.07	0.027	336 (1.09)	1 (2.38)	2.20	0.30 - 16.07	0.435
High blood sugar findings	221(0.88)	25 (1.13)	1.28	0.85 - 1.95	0.235	246 (0.91)	0	-	-	-
BMI									
Underweight (reference)	477 (1.20)	40 (1.27)	1.06	0.76 - 1.46	0.732	516 (1.26)	1 (1.85)	1.55	0.21 - 11.22	0.664
Normal weight	13,005 (32.67)	1,023 (32.41)	0.99	0.91 - 1.07	0.67	14,003 (32.64)	25 (46.30)	1.78	1.04 - 3.04	0.035
Overweight	10,396 (26.12)	898 (28.45)	1.13	1.04 - 1.22	0.004	11,283 (26.30)	11 (20.37)	0.72	0.37 - 1.39	0.325
Obesity	3,323 (8.35)	316 (10.01)	1.22	1.08 - 1.38	0.001	3,634 (8.47)	5 (9.26)	1.10	0.44 - 2.77	0.835
Physical disability	380 (1.52)	62 (2.81)	1.86	1.43 - 2.46	<0.001	442 (1.63)	0	-	-	-
Regular intake of blood pressure medications	2,226 (8,90)	304 (13.79)	1.64	1.44 - 1.86	<0.001	2,528 (9.30)	2 (6.90)	0.72	0.17 - 3.03	0.657
Regular intake of medications	3,618 (14.47)	500 (22.69)	1.73	1.56 - 1.93	<0.001	4,110 (15.12)	8 (27.59)	2.14	0.95 - 4.83	0.068
Pregnancy or delayed menstrual cycle	233 (0.87)	12 (0.53)	0.60	0.34 - 1.08	0.089	245 (0.85)	0	-	-	-
Discouragement, depression, or little hope (past 30 days)	888 (3.55)	117 (5.31)	1.52	1.25 - 1.86	<0.001	1,000 (3.68)	5 (17.24)	5.45	2.08 - 14.32	<0.001
Little interest or enjoyment in the past 30 days	660 (2.64)	92 (4.17)	1.61	1.29 - 2.01	<0.001	748 (2.75)	4 (13.79)	5.65	1.96 - 16.27	<0.001
Family history of diabetes	4,389 (17.55)	450 (20.42)	1.21	1.08 - 1.34	0.001	4,834 (17.78)	5 (17.24)	0.96	0.37 - 2.52	0.399
Behaviors/lifestyle/habits									
Cigarette consumption	1,038 (3,66)	108 (4.52)	1.25	1.02 -1.53	0.032	1,144 (3.73)	2 (4.76)	1.29	0.31 - 5.35	0.724
Use of psychoactive substances	15 (0.08)	3 (0.17)	2.20	0.64 - 7.61	0.213	17 (0.08)	1 (4.55)	59.03	7.51 -463.93	<0.001
Alcohol consumption	1,249 (6.29)	73 (4.09)	0.63	0.50 - 0,81	<0.001	1,321 (6.11)	1 (4.76)	0.77	0.10 - 5.73	0.798
Fruit and vegetable consumption	23,060 (92.68)	2,049 (93.48)	1.13	0.95 - 1.35	0.167	25,082 (92.74)	27 (96.43)	2.11	0.29 - 15.57	0.462
Physical activity	15,264 (63.91)	1,285 (61.07)	0.89	0.81 - 0.97	0.010	16,531 (63.68)	18 (62.07)	0.93	0.44 - 1.98	0.857
Condom use	5,190 (18.30)	408 (17.09)	0.92	0.82 - 1.02	0.143	5,592 (18.21)	6 (14.29)	0.75	0.32 - 1.78	0.512
BMI									
Underweight	477 (1.20)	40 (1.27)	1.06	0.76 - 1.46	0.732	516 (1.26)	1 (1.85)	1.55	0.21 - 11.22	0.664
Normal weight	13,005 (32.67)	1,023 (32.41)	0.99	0.91 - 1.07	0.767	14,003 (32.64)	25 (46.30)	1.178	1.04 - 3.04	0.035
Overweight	10,396 (26.12)	898 (28.45)	1.13	1.04 - 1.22	0.004	11,283 (26.30)	11 (20.37)	0.72	0.37 - 1.39	0.325
Obesity	10,396 (26.12)	316 (10.01)	1.22	1.08 - 1.38	0.001	3,634 (8.47)	5 (9.26)	1.10	0.44 - 2.77	0.835

HIV: Human Immunodeficiency Virus. AIDS: Acquired Immunodeficiency Syndrome. BMI: Body Mass Index.

In the multivariate analysis, the variables that were statistically significant in the bivariate analysis were included, and a model was constructed using those that were significantly associated with having an all cause hospitalization, which were having a history of heart disease, respiratory disease, chronic kidney disease, and cancer, regular intake of any medication, and smoking. The only variable that appears to be associated with a lower risk of all-cause hospitalization is alcohol consumption. Concerning hospitalizations for psychiatric reasons, the multivariate analysis indicated that having felt discouraged, depressed, or hopeless in the past 30 days and regular intake of medication were associated with psychiatric hospitalization and having normal weight. No variables were identified that were associated with a lower risk of psychiatric hospitalization (Table 3).

**Table 3:** Multivariate analysis of variables associated with all-cause and psychiatric hospitalizations.

Variable	OR	95% CI	P
All-cause hospitalization*			
Cigarette consumption	1.44	1.12 - 1.85	0.004
Personal history of heart disease	1.71	1.33 - 2.20	<0.001
Personal history of respiratory disease	1.30	1.04 - 1.61	0.019
Personal history of chronic kidney disease	1.66	1.13- 2.45	0.010
Personal history of cancer	1.65	1.14 - 2.40	0.009
Regular intake of medications	1.35	1.17 - 1.56	<0.001
Alcohol consumption	0.67	0.50 - 0.84	0.001
Age (Reference: year)	1.01	1.00 - 1.01	<0.001
Sex (Reference - male)	0.94	0.85 - 1.05	0.257
Hospitalization for psychiatric reasons**			
Discouragement, depression, or little hope (past 30 days)	5.12	1.89 - 13.87	0.001
Little interest or enjoyment (past 30 days)	2.84	1.10 - 7.31	0.031
Age (year)	0.98	0.96 - 1.01	0.159
Sex (Reference- Male)	1.76	0.84 - 3.70	0.133
BMI Normal weight	3.13	1.40 - 6.98	0.005

*R2= 0,0139 ** R2= 0,0514 (p <0,05).

## Discussion

The present study made it possible to identify those variables that, when asked at the time of enrollment in an EAPB (also known as a health insurance company), showed a statistical association with post-enrollment hospitalizations: For all-cause hospitalization, reporting a history of heart disease, respiratory disease, chronic kidney disease, cancer, regular intake of any medication, and smoking; and for hospitalization for psychiatric reasons, it was found that feeling discouraged, depressed, or hopeless in the past 30 days and regular intake of any medication were associated with this outcome. Although the results do not provide a model that explains hospitalizations with the variables described above, primary care providers and the insurance company need to identify the profiles of members who may be more likely to be hospitalized.

In summary, the above findings are particularly relevant to the EAPB because it can prioritize these members with such risks and manage them early to prevent catastrophic events such as preventable hospitalizations. As a result, they provide information for both the insurer and the primary care provider to conduct analyses to redirect programs that need to be strengthened to meet the identified needs of the insured population. Taking into account the above, and from the perspective of insurance within the General Social Security Health System, the EAPBs are responsible for managing the health risks for their members and intervening promptly in the risks of their insured population, as well as articulating services that ensure effective access to quality services^13^. Therefore, predictive analysis like the one shown here is important for timely intervention at the insurance and primary care levels. Although each insurance company must conduct an annual characterization of its members and report it to the health authorities^14^, this does not allow for rapid management of individual risk, nor does it identify priorities from the moment of enrollment, as was done in this analysis.

In addition, appropriate risk management would reduce the costs of preventable health events and optimize the resources allocated to health promotion and maintenance programs and the prevention of disease and its complications. Moreover, and recognizing the finiteness of resources in the healthcare system and understanding its business model, if the risks of the enrollees are not managed, if there are more claims (such as the hospitalizations analyzed in this study), and ifthe expenses incurred, added to the other expenses of insurers, exceed the value of the money received, the EAPB will inevitably incur losses^15^. However, analyses such as the one presented in this study make it possible to make predictions based on individual data and reduce the probability of occurrence of preventable events through appropriate planning of the health service delivery system^16^. In addition to health risk management, this analysis makes it possible to anticipate and prevent not only health complications for the individual, but also complications that are costly to the system^17^.

It is still recommended in clinical practice and the insurance industry to conduct individual health risk assessments that emphasize the promotion of health and prevention of disease in individuals and populations using information from their medical and family medical history. The assessments are done with the aim of early identification of individual risks for chronic and hereditary diseases, which leads to the reinforcement that activities at the time of enrollment, such as the survey shown in this study, would allow for better members’ risk management^18^.

It should be noted that diseases such as high blood pressure and diabetes, precursors of chronic kidney disease (CKD), show that it is still necessary to prioritize strategies to prevent the progression of the latter since data show that 33.4% of patients with hypertension or diabetes have not been screened to determine the presence or absence of CKD^19^. In this sense, this study found that CKD is associated with all-cause hospitalizations, which reinforces the need to apply secondary prevention strategies in the population at risk and identify CKD in its early stages.

Modifiable health risk behaviors have also been shown to affect healthcare expenditures, including depression, stress, elevated blood sugar levels, extremely high or low body weight, tobacco smoking, high blood pressure, and sedentary lifestyle, which are associated with risks of poor health outcomes and higher expenditures^20^ like those in this study. In addition, the characterization survey presented is similar to the Individual Health Risk Assessment (IHRA) and both are tools that serve to emphasize health promotion and disease prevention in individuals. IHRAs are used in healthy individuals or in the presence of identifiable risk factors to estimate the risk of developing disease^17^. In this case, the survey used made it possible to estimate hospitalization risk, reinforcing the need to use these tools in risk management in health insurance.

The present study included factors such as eating behaviors and consumption of alcohol, tobacco, and other substances that, although not associated with an increased risk of hospitalization, are not usually included in predictive models of hospitalization risk reported in the literature^21^^,^^22^. In addition, our study included data such as BMI, which has been reported to be important in hospital care, and some studies have found that low weight was associated with a higher risk of hospitalization^23^. Although this study found a higher risk in patients with normal weight, it is important to remember that the data were self reported by the patients, and the instrument was not validated. In addition, studies have shown that certain sociodemographic variables, such as socioeconomic status, marital status, race, food insecurity, social isolation, and medication insecurity, are associated with an increased risk of hospitalization^24^^,^^26^. Other information provided by patients that could contribute to better stratification, such as that collected by the characterization tool described in this study, are data such as quality of life and behavioral or social factors^27^.

The main limitations of this study include the self-report nature of the characterization survey, which may introduce bias into the information obtained and the fact that it was not previously validated. In addition, the survey was not administered to all members for the reasons described above, and we do not have data on all insured individuals for the period analyzed. Similarly, the positive association found between alcohol consumption and lower risk of hospitalization may be due to measurement bias, as the instrument used to obtain the data was not previouslyvalidated. In addition, we did not use instruments recommended for measuring alcohol consumption, such as AUDIT, ASSIST, or CAGE according to the recommendations of the Ministry of Health and Social Protection^28^. Finally, we acknowledge as a major limitation of the study that the likely confounding effect of the COVID-19 pandemic was not accounted for in the analysis presented in this article.

A validation of the characterization survey is recommended for future analyses, as well as incorporating variables associated with higher risk of hospitalization outcome identified in other studies.

## Conclusions

As described above, the characterization of health insurance applicants at the time of enrollment provides useful information for insurance and primary care decision-makers to intervene early in members’ risks and prevent health events that require hospitalization. In this way, the optimization of resources and better health outcomes are achieved, which would be reflected in the viability of insurance companies.
